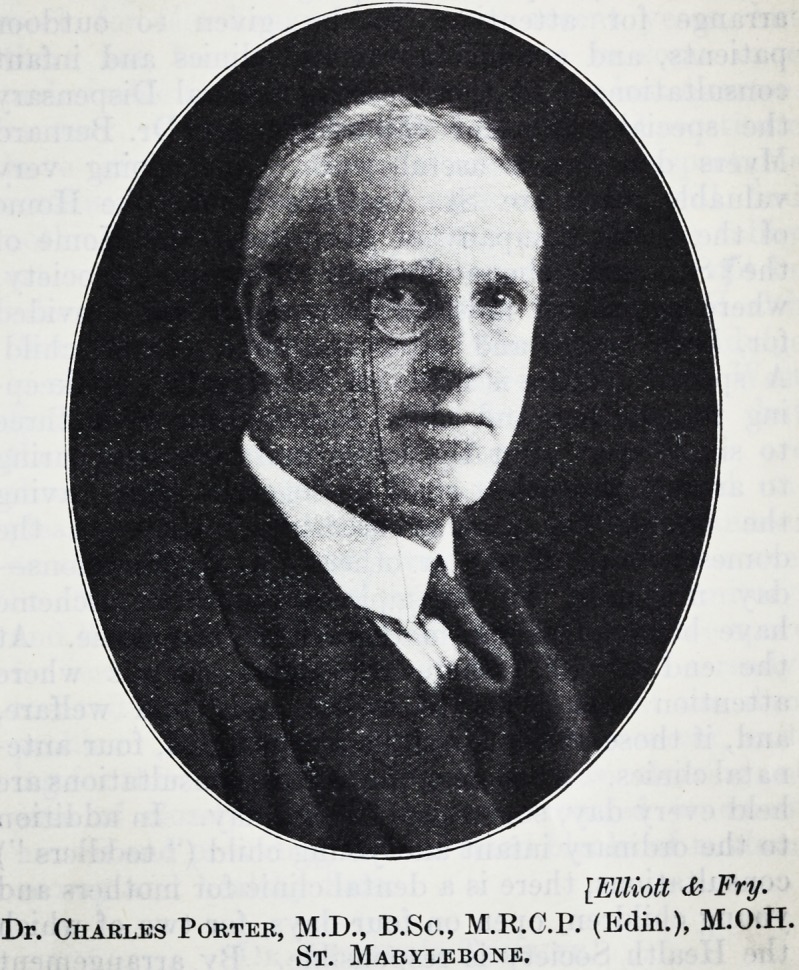# The Public Health: Interviews with Local Authorities: Marylebone

**Published:** 1924-09

**Authors:** 


### THE PUBLIC HEALTH.

### INTERVIEWS WITH LOCAL AUTHORITIES.

### XIX.—THE METROPOLITAN BOROUGH OF ST. MARYLEBONE.

In Dr. Charles Porter, the Borough of St. Marylebone is fortunate in having a Medical Officer of wide outlook who can bring to bear on his own local problems a judgment and an experience in the field of public health which find expression in a variety of ways. Dr. Porter, who is a barrister as well as a Doctor of Medicine, is well known both as a writer and lecturer. He very courteously gave us an account of the work more immediately to his hand in this important metropolitan borough. The interview was arranged at short notice in the holiday season, and the Chairman of the Health Committee was, unfortunately, unable to be present.

[Elliott & Fry.

Alderman Alfred C. Rickatson, Chairman St. Marylebone Public Health Committee.

[Elliott & Fry.

Dr. Charles Porter, M.D., B.Sc., M.R.C.P. (Edin.), M.O.H. St. Marylebone.

#### Two Populations.

Though we speak with diffidence on so delicate a subject, Marylebone, we believe, may fairly claim to be the premier metropolitan borough. When we consider the work of a London borough we have to think of quite different standards from those which we should apply in the case of a provincial town. There are, for example, two populations in Marylebone. There is the population of the census, which records the people who sleep in the borough ; this numbers 105,000. But the people who flock into it in the morning and out again in the evening, who live a very important part of their lives within the boundaries of St. Marylebone, are a million rather than a hundred thousand. The sleeping population grew from 60,000 in 1801 to 160,000 in 1871, and in the subsequent half century had fallen to 105,000. In the case of a provincial borough we should say that such a drop was evidence of decline in status and importance. In Marylebone it corresponds, on the contrary, with an enormous growth and rise in importance. As a direct indication of this, we were informed that the gross yearly valuation of rateable property had risen from £1,400,000 in 1871 to £3,000,000 in 1921. As showing the progress which, thanks to the Health Committees and their Medical Officers, and to the advance of public opinion in the domain of health, has been recorded, we may allow ourselves to quote one or two of the impressive figures furnished to us by Dr. Porter. The death rate fell from 24.16 to 12.87, the infantile mortality rate from 173 to 66, measles from 40 to nil, whooping cough from 136 to 7, diarrhœa from 196 to 22. There were 1,200 victims of the loathsome small-pox in 1871, of which 238 proved fatal ; in 1921 there was not a case. These are not facts of a kind to give pause to the hustling millions who make their way from Marylebone and Baker Street Stations to Wembley with the idea of visualising something of the wonders of the British Empire. It is too much to expect them to reflect that such matters have an important bearing on the fact of the Empire itself. But the public health student and worker will see in these figures something for deep satisfaction. The stately Town Hall of Marylebone is an embodiment of the spirit of progress in the borough ; that progress would be impossible without the constant vigilance of the Health Committee and their Medical Officer and his staff. Let us look at some of the questions which concern the resident population.

### The Mothers and the Toddlers.

In regard to the Maternity and Child Welfare work, Dr. Porter laid particular stress on the debt that the Marylebone public owe to the voluntary worker. The scheme contains both voluntary and official elements. Of the voluntary elements the Council and the inhabitants of the borough are in particular indebted to the St. Marylebone Health Society and those who work for it for what they have done and what they are doing in relation to maternity and child welfare. There are other bodies that assist, and are more or less definitely affiliated to the main scheme, the connecting link being the Public Health Department of the Council. For instance, Queen Charlotte's Hospital and Middlesex Hospital, in addition to providing lying-in accommodation, arrange for attention to be given to outdoor patients, and conduct ante-natal clinics and infant consultations. At the Western General Dispensary the special clinic for children under Dr. Bernard Myers does most useful work. Also doing very valuable work are St. Agatha's Home, the Home of the Little Company of Mary, and the Home of the St. Marylebone Female Protection Society, where unmarried mothers particularly are provided for, both before and after the birth of the child. A special feature is made at St. Agatha's of keeping the mother and child together for from three to six months after the birth, and of endeavouring to arrange that they shall be together after leaving the home. On several occasions places on the domestic staff of one or other of the institutions—day nurseries, for example—within the scheme have been found for mothers from the home. At the end of 1923 there were nine centres where attention was given to infant and child welfare, and, if those at the hospitals are included, four ante-natal clinics. At some of the centres consultations are held every day, sometimes twice a day. In addition to the ordinary infant and young child (" toddlers ") consultations, there is a dental clinic for mothers and young children, open on four days, for two of which the Health Society is responsible. By arrangement with the London County Council the dental clinic is used also by school children, and in conjunction with this there is likewise a minor ailment treatment centre for school children.

### The School Child—An Unprofitable System.

When the toddlers arrive at school age, they pass from the care of the Medical Officer of Health. Dr. Porter pointed out that the medical officer of a Metropolitan Borough is not school medical officer. The work in connection with both schools and school children is entirely in the hands of the London County Council and their officers, and in no part of the country is the divorce between the general public health work and school medical work so complete as in London. It is impossible to see any justification for it on public health or economic grounds, and it is difficult to understand why either the central government authorities or the local authorities have for so long acquiesced in the arrangement. In regard to infectious diseases as they affect the school child, the fact that there are two staffs engaged generally leads to the expenditure of a great deal of energy as a result of each endeavouring to keep the other informed as to the state of affairs. So far as the general health of the school children is concerned, the local medical officer of health receives no direct information; if he desires to learn where inspections are to be made, he must ask to be informed or find out from the weekly gazette. Generally, it may be said that there is some duplication of work, a quite unnecessary amount of correspondence, and from time to time cross-purposes, all of which is contrary to the best interests of the public health locally and particularly to those of the children.

### Tuberculosis.

There is an effective Dispensary scheme in operation in St. Marylebone under the control of Dr. J. D. Saner. The provision of Sanatorium and Hospital accommodation is within the functions of the London County Council and the Metropolitan Asylums Board. Dr. Porter described the relations with those authorities for the co-ordination of treatment and continuity of records as excellent. Dr. Porter drew our attention to some observations of Dr. Saner's on an interesting and saddening feature of the work of putting the tuberculous patient on his legs, which are well worth reproduction. He says that the difficulty of finding employment for the consumptive person still remains acute. Employers are loath to employ a man, first, on account of infection, and secondly, because he is not a 100 per cent. unit. Now there is no necessity for any consumptive to be infectious to his fellow-workers if he takes an ordinary amount of care. If he has been to a sanatorium, he has received full instructions as to how to avoid being a danger to others, and if thereafter he carries out what he has learnt, the chance of infection is reduced to a minimum.

### The Man with the Sputum Flask.

The man who coughs and expectorates is provided with a sputum flask in which to spit; this, unfortunately, is enough to damn him in the eyes of his neighbours, and people in the street give him a wide berth. These same people, however, have no such terror of the man who spits on the pavement, in tram, 'bus or train, and is the real source of danger to the community—they tolerate him, but the poor fellow who is doing his best not to infect others with his complaint is ostracised. There is no reason why a man should not return to his original employment if the conditions under which he works are suitable; too much stress has been laid upon the idea that it is an absolute necessity for him to work out of doors. Mr. Ford, of motor car fame, can find employment for all disabled men in his works even if they can do only a few hours' work each day. It seems a pity that the big firms in our country cannot do likewise.

### The Case of the Million.

The larger of the two populations to which we have referred is intimately concerned, however little the individual units may realise it, with the provision made for its welfare by the Health Committee—pure water, good drainage, public sanitary conveniences, the removal and disposal of house refuse, and all the hundred and one matters that make up the sanitary care of the district. This large in-and-out population is, perhaps, especially concerned with questions governing food supply and workshop inspection. There are 3,576 workshops on the register in the borough, and there are, too, large numbers of houses where outworkers are engaged. Dr. Porter was only too ready to recognise that the big West End stores need practically only formal supervision in medical and sanitary matters. That this is so is a striking testimony to the advance in public opinion generally and to the more enlightened views of employers of labour which is so salutary a feature of the last half-century.

### Premises that should be Registered.

The number of premises in the Borough in which meals are provided or food is sold ready cooked or is prepared for sale is very large. At the end of 1923 there were 232, this number including restaurant, dining room and coffee shop kitchens, 172 ; tea-rooms and pastry-cooks, 40 ; hotel kitchens, 20 ; fried fish shops, 24 ; and fish-curers, 5. In addition, there were a number of shops in which meat, ham, sausages, etc., were cooked and sold only over the counter. On more than one occasion the Borough Council, at the suggestion of the Public Health Committee, have directed the attention of the Ministry of Health and the London County Council to the necessity for legislation requiring registration of food premises of this class. Up to the present no step in this direction has been taken by either of the authorities mentioned. There is routine visiting of a number of restaurants, etc., but, in addition, during the year 1923, a special investigation was made with the object of discovering exactly the arrangements provided in restaurants, etc., for the sanitary convenience of the employees, and particularly the provision made for and the instructions given as to the washing of hands after using the conveniences and at other times. Some unsatisfactory features were brought to light, but the Medical Officer noted with pleasure the readiness with which the persons responsible for finding a remedy recognised the need for improvement, and took steps to provide it.

### The Saturday Night Food Stall.

There are considerable numbers of food stalls in the market streets. Definite arrangements are made for the keeping of such as are used for the sale of food under close observation. Throughout 1923 visits were paid to all the market streets every day, a special feature being made of Saturday night and Sunday morning inspections.

### Food Handling.

Dr. Porter looks forward to the day when the Health Authorities will be given powers to deal with offences against hygiene in the handling of food. In this respect he says that we have much to learn from American methods. Of the more progressive nations, we are perhaps the most neglectful in this matter. We ought not to view with complacency the open cart piled high with fresh carcases serving as a couch for the porter with the dirty clothes ; nor the open butcher's slabs, where the dust and the flies are active ; nor the woman with the filthy hand who fingers all the stock before she makes her decision ; nor the boy who recovers the loaf from the mud and wipes the dirt from it with his already dirtier sleeve.

### The Changing Character of the District.

We have referred to progress in this district of St. Marylebone, which is in marked contrast with the decline in the sleeping population. Possibly this is most familiar and most striking in the case of the great stores on the north side of Oxford Street. In passing, there is a grim reminder of earlier days at Marble Arch Corner, where stood the hangman's tree of Tyburn. There are areas between Oxford Street and Hampstead, where lies the northern boundary of the borough, which have provided work for the hangman in more recent years, but there has been a wonderful change in some once undesirable neighbourhoods even in the last quarter-century. Unsavoury haunts of prowler and prostitute have given place to most eminently respectable institutions, where after sundown you will find nothing more disturbing than a peace-loving tabby cat curled up on the front doorstep. It must not be inferred that all is well and that the Public Health Committee and their Medical Officer can also indulge in sleep. True, their Harley Street and their West End Stores are happily free from reproach ; but there are mean shopping places, and there are slums, if you look for them, very dingy and unpleasant places, awaiting the housebreaker's hammer as soon as we can get away from the Geddes axe. St. Marylebone is fast ceasing to be a dormitory, and it may be that in the demand for valuable space for institution and workshop will be found a ready-to-hand and possibly complete solution of the slum problem. The Borough Council are not, however, waiting for this, and at the moment are busy clearing one area of closely packed and overcrowded tenement houses and providing in their stead blocks of modern, roomy and healthy flats.

### Dr. Porter's Tribute.

Dr. Porter, in expressing his indebtedness to the Chairman of the Health Committee, Mr. Alderman Rickatson, and his predecessor in the chair in 1923, Mr. Alderman J. Fettes, for their support generally, emphasised the fact that this was no formal acknowledgment. It was largely owing to the view taken of his request for leave of absence by the Committee and by the two gentlemen who were in the chair that the Council allowed Dr. Porter to accept the invitation of the Health Section of the League of Nations to visit the United States and study the system of public health administration in vogue there. (We hope to make further reference to this.) During his absence he had not one moment of anxiety so far as the work of the department was concerned, and on his return after nearly five months' travel he found all matters in perfect order. This was no less due, said Dr. Porter, to the Committee, their interest in the work and their willingness to assist the workers, than to the work of a loyal and efficient staff. Dr. Jameson, the Deputy Medical Officer of Health, took Dr. Porter's place during his absence and devoted himself unsparingly to the work.

## Figures and Tables

**Figure f1:**
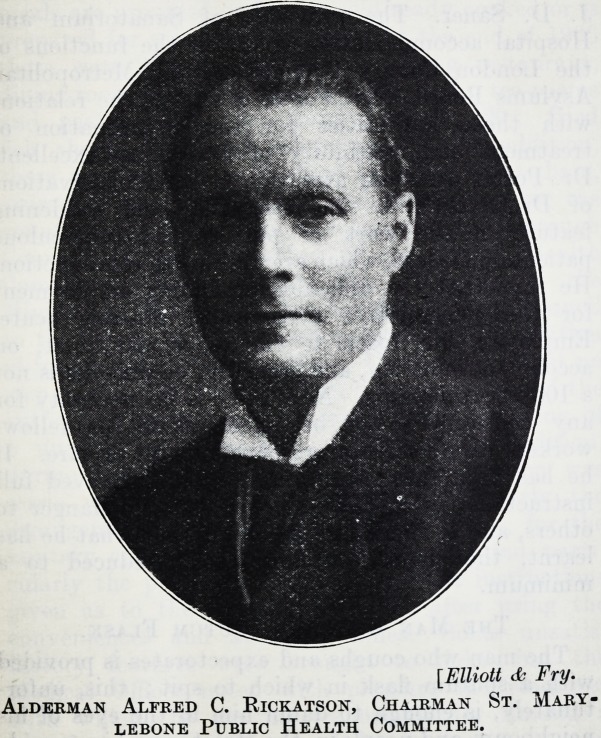


**Figure f2:**